# Medical treatment of mammary desmoid-type fibromatosis: which benefit?

**DOI:** 10.1186/s12957-017-1148-x

**Published:** 2017-04-18

**Authors:** Louise Scheer, Massimo Lodi, Sébastien Molière, Jean-Emmanuel Kurtz, Carole Mathelin

**Affiliations:** 1 Unité de Sénologie, Hôpital Hautepierre, Hôpitaux Universitaires de Strasbourg, CHRU, 1 Avenue Molière, 67200 Strasbourg, France; 2 Unité d’Imagerie de la Femme, Hôpital Hautepierre, Hôpitaux Universitaires de Strasbourg, CHRU, 1 Avenue Molière, 67200 Strasbourg, France; 3 Service d’Oncologie, Hôpital Hautepierre, Hôpitaux Universitaires de Strasbourg, CHRU, 1 Avenue Molière, 67200 Strasbourg, France; 4 Institut de génétique et de biologie moléculaire et cellulaire, IGBMC - CNRS UMR 7104, INSERM U964, Université de Strasbourg, Illkirch, France; 5 Centre Hospitalier de Sarrebourg, Rue des Roses, 57400 Sarrebourg, France

**Keywords:** Desmoid-type fibromatosis, Extra-abdominal fibromatosis, Breast fibromatosis, Tyrosine-kinase inhibitors, Sunitinib, Wnt-beta catenin

## Abstract

**Background:**

Breast fibromatosis is a rare disease characterized by monoclonal fibroblast proliferation. It has no ability to metastasize but has a high local recurrence rate and often infiltrates surrounding tissues. Surgical treatment is the reference, but recently, new targeted therapies have emerged. We report an original case of a patient with breast fibromatosis who received exclusive medical treatment. Our aim was to analyze these treatments based on the clinical and radiological outcome, iatrogenic effects, and pharmacological action.

**Case presentation:**

We report the case of a 19-year-old woman who developed a desmoid-type fibromatosis of the lower inner quadrant of the right breast, measuring 50 × 25 mm (i.e., a volume of 27.4 cm^3^). Initial surgery was not possible because of potential esthetic and functional prejudice. Thus, she had an exclusive medical treatment including several lines: NSAIDs with tamoxifen and triptorelin, followed by sorafenib, then interferon α2b, and finally sunitinib. With tyrosine-kinase inhibitors (TKIs) (sunitinib), a significant partial response was observed (57% reduction of the maximal tumoral volume). For each treatment, we provided the clinical and radiological outcome in association with known pharmacological action.

**Conclusions:**

TKI had been an interesting alternative option to initial surgery, providing at least a partial response and potentially allowing less mutilating surgery. However, no pharmacological mechanism can unequivocally explain TKI efficacy. In general, breast fibromatosis should be treated along with oncologist and interventional radiologists in a trans-disciplinary modality, thus offering an adapted treatment for this particular desmoid-type fibromatosis localization.

## Background

Fibromatoses (formerly desmoid tumor) are clonal fibroblast proliferations that develop in the deep soft tissue. One of their characteristics is their tendency to local recurrence, without the ability to metastasize. These lesions are usually poorly confined and infiltrate the surrounding tissues. Fibromatoses are classified into three groups according to the WHO: fibromatosis of the abdominal wall (AF), extra-abdominal (EAF), and intra-abdominal (IAF) [1].

IAF is linked to familial adenomatous polyposis while both AF and EAF often occur sporadically. Etiology of these lesions remains uncertain: genetic mutations, trauma, hormonal factors, etc., have been mentioned. The incidence of sporadic fibromatosis (AF and EAF) ranges from two to four cases per million people [2–4]. EAF are predominant in women (ratio 2:1), and the average age of onset is 37 years [5]. In terms of localization, EAF may involve the trunk (47.2%), the extremities (33.7%), the head (10.9%), or other sites (8.1%) [5].

Clinically, breast fibromatosis presents as a palpable, firm mass that may adhere to the chest wall, sometimes associated with skin retraction. According to the French National College of Gynecologists and Obstetricians (CNGOF), there is neither sufficient data to recommend surgery over conservative treatment nor optimal follow-up modalities and timing [6]. The overall recurrence rate after surgery ranges from 18 to 39% [7–11]. Local recurrence rate after surgery with complete resection is 7–28% [7, 8, 10, 12–16] and 26–100% with incomplete resection [7, 8, 10, 13, 14]. Moreover, surgery may have functional and esthetic consequences.

Before 2000, most breast fibromatoses were surgically removed. Better understanding of the biology of these tumors and the introduction of new drugs (sunitinib (Sutent®), sorafenib (Nexavar®)) have enabled the development of medical protocols using targeted therapies. Few clinical studies evaluated targeted therapies efficacy in EAF; consequently nowadays, no guidelines are available.

We report an original case of a patient with breast fibromatosis who received exclusive medical treatment. Our aim was to analyze these treatments based on the clinical and radiological outcome, iatrogenic effects, and pharmacological action, as an alternative to initial surgery.

## Case presentation

In October 2012 at the age of 19, Ms. L.E., nulliparous, with no previous medical history, was examined for breast pain and lump in the lower inner quadrant of the right breast. She reported that the mass appeared in 2009 and has slowly grown in size. Clinical examination confirmed the presence of a hard, ill-defined mass involving the pectoral muscle, associated with skin retraction. There was no suspicious axillary node.

**Fig. 1:**
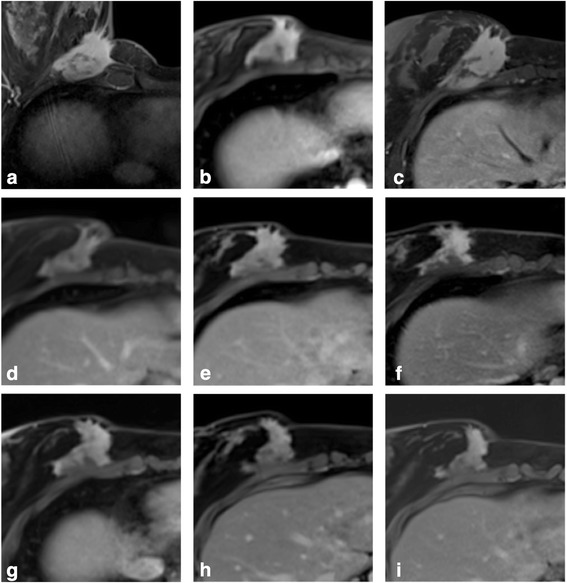
Tumor evolution on MRI. **a**–**c** MRIs during tamoxifen + arthrocine association (tumoral volume respectively 27.4, 27.1, and 30.4 cm^3^). **d**–**f** MRIs during sorafenib (tumoral volume respectively 24.7, 26.5, and 25.6 cm^3^). **g** MRI during IFN (tumoral volume 26.4 cm^3^). **h**, **i** MRIs during sunitinib (tumoral volumes respectively 15.3 and 13.2 cm^3^)

Mammography and breast ultrasound revealed a heterogeneous, partially well-limited mass. MRI confirmed the presence of a mass infiltrating the lower part of the major pectoralis muscle, measuring 50 × 25 mm in size and 27.4 cm^3^ in volume (Fig. 1a).

Core needle biopsy sample analysis showed proliferation of fibroblastic-like and/or myofibroblastic-like spindle cells, arranged in moderately rich collagen-dense arrays. Mitosis was rare. The proliferation included striated muscle fibers. There was no necrosis. Immunohistochemical analysis showed negativity of anti-pan keratin antibodies, estrogen receptors (ER), protein S100, CD34, calretinin, CD117, and p53. Some cells were expressing smooth muscle α-actin, and nuclear staining with anti β-catenin antibody was noted. Ki67 was estimated at 5%. These results confirmed the diagnosis of breast fibromatosis. After multidisciplinary discussion, we opted for an initial medical treatment, because the depth of muscular involvement increased esthetic and functional risks of surgery.

As a first-line treatment, the patient received non-steroidal anti-inflammatory drugs (NSAID; arthrocine, 200 mg orally per day) plus tamoxifen (40 mg orally daily), under cover of a GnRH agonist (long-acting triptorelin 3.75-mg intramuscular injection every 28 days), to prevent the risk of ovarian cysts linked to tamoxifen. In the months following the initiation of treatment, stabilization of tumoral volume and decrease in skin retraction were observed. Breast MRI at 5 months showed stable tumoral size (volume 27.1 cm^3^, Fig. 1b). The same treatment was pursued until disease progression at 9 months (volume 30.4 cm^3^, Fig. 1c) and was then replaced by a tyrosine-kinase inhibitor (sorafenib, 400 mg per day orally). After 10 days, the daily dose of sorafenib was reduced to 200 mg due to a grade 2–3 palmoplantar erythrodysesthesia. After 4 months of sorafenib, both clinical examination and MRI showed significant decrease in tumor volume (24.7 cm^3^, Fig. 1d). Sustained response was still obtained at 1 year of treatment (volume 26.5 cm^3^, Fig. 1e). At 1.5 years due to cutaneous toxicity and tumor stagnation (volume 25.6 cm^3^, Fig. 1f), sorafenib was replaced by interferon α2b (five subcutaneous injections of 6 million IU per week). Due to severe asthenia and tumor progression at the MRI 3 months after (volume 26.4 cm^3^, Fig. 1g), interferon was stopped. Sunitinib (25 mg a day) was then introduced, but cutaneous toxicity associated with constipation, led to intermittent administration during periods of 10 to 15 days to decrease toxicity. Eight months later, MRI tumor volume was 15.3 cm^3^ (Fig. 1h) and 13.2 cm^3^ at 13 months (decrease of 57% compared with the maximum tumoral volume, Fig. 1i). Currently, the patient is receiving the same treatment, but side effects similar to those previously observed impair the quality of her life (Table 1). Therefore, and because the patient is now planning a pregnancy, removal by surgery or cryotherapy is now being considered.

**Table 1:** Tumor size evolution on breast MRI

IRM date	Tumoral size (mm) max/min	Tumoral volume (cm^3^)	Evolution^a^ (%)	Medical treatment period/drug(s)	
26/10/12	50 × 25	27.4	90.1	22/11/2012–02/07/2013	Tamoxifen 40 mg/day + arthrocine 200 mg/day
18/03/13	50 × 25	27.1	89.1		
18/06/13	54 × 26	30.4	100.0		
28/11/13	51 × 18	24.7	81.3	02/07/2013–05/09/2013	Sorafenib 400 mg/day
27/06/14	50 × 22	26.5	87.2	05/09/2013–15/01/2015	Sorafenib 200 mg/day
12/12/14	49 × 21	25.6	84.2		
21/04/15	49 × 23	26.4	86.9	15/01/2015–09/03/2015	Interferon α2b 5 × 6 10^6^ UI/week
				09/03/2015–20/05/2015	Interferon α2b 5 × 6 10^6^ UI/week + arthrocine 200 mg/day
14/01/16	32 × 21	15.3	50.3	30/05/2015–now	Sunitinib 25 mg/day by periods of 10 to 15 days
01/07/16	31 × 18	13.2	43.4		

^a^Tumoral volume evolution compared to the maximal tumoral volume

## Discussion

It is accepted that surgery is still the standard of care for mammary fibromatosis, and wide local excision is recommended. Alternative treatments, such as radiotherapy, are usually proposed for patients experiencing multiple recurrences [17]. Radiotherapy can lead to growth arrest but side effects such as pain, limb edema, and skin toxicity can appear. Because of our patient’s young age and the potential adverse effects, we decided that radiotherapy was not indicated as a first-line treatment. The exclusive medical treatment that our patient underwent allowed reduction of more than half of the volume of the tumor. Clinical efficacy, side effects, and pharmacological mechanisms of breast fibromatosis medical treatment are discussed below.

Hormone therapy with tamoxifen and GnRH analogs failed to show any antitumoral activity in our case. Some studies have indicated a beneficial effect of tamoxifen either alone [18–21] or in combination with NSAIDs [22, 23] in non-mammary EAF. Mammary fibromatosis usually do not express ER [24]. However, one case report of a patient with breast fibromatosis, negative for hormone receptors (estrogen and progesterone), showed a significant decrease in tumor size with tamoxifen at a daily dose of 20 mg for 14 months [25]. In this case, the beneficial effect of tamoxifen was attributed to direct cytotoxic effect or inhibition of the Wnt/β-catenin pathway.

NSAID action is related to the Wnt/β-catenin pathway, via cyclooxygenase-2 (COX-2). In our case, immunohistology study showed intranuclear accumulation of β-catenin, which may be present in up to 82% of breast fibromatosis [26]. The Wnt signaling pathway involving β-catenin as co-activator plays a major role in the pathophysiology of fibromatosis. Mutations in the CTNNB1 gene, encoding for the β-catenin, have been reported in the EAF in up to 75% of the cases (in a study involving 145 patients) [27]. In healthy cells, Wnt proteins bind to a receptor complex consisting of Fz and LRP6 (low-density lipoprotein receptor-related protein 6) proteins. This binding is regulated by the LRP6 phosphorylation by two kinases, GSK3 and CK1γ. At rest, these two kinases phosphorylate β-catenin, leading to its ubiquitylation and destruction by the proteasome [28]. Mutations of β-catenin in tumoral cells may prevent this phosphorylation, leading to β-catenin accumulation and translocation to the nucleus to activate transcription of target genes, in particular the one of COX-2 [29]. COX-2 promotes tumor growth (inhibition of apoptosis, stimulation of angiogenesis, migration, and cell proliferation) by increasing the expression of growth factors [29]. Use of NSAIDs, which are COX-1 and 2 non-selective inhibitors, is based on this rational. In addition, one study suggests that COX-2 is involved in the painful symptoms of fibromatosis, via its secretion by mast cells of the microenvironment, which may explain the clinical benefits of NSAIDs [30]. However, in our case, the treatment with tamoxifen and arthrocine did not allow a reduction in tumoral volume.

Interferons (IFNs) are cytokines secreted by leukocytes (IFN-α) and fibroblasts (IFN-β). Once bound to their receptor (IFNAR-1), they activate in particular the JAK/STAT pathway that regulates expression of response genes having antiproliferative functions. Several cases of complete remission upon treatment with IFN-α have been reported in patients affected by limbs [31, 32] and pelvic [33] fibromatosis. Partial response has been observed in a patient with temporal fossa [34] and foot [35] fibromatosis. A study showed that IFN signaling is regulated by the β-catenin pathway [36]. IFN-α therapy did not allow tumoral response, which may be explained by experimental evidence. According to Tjandra et al., IFN may decrease the proliferation of fibromatosis tumor cells, but do not affect tumoral stem cells, which may increase their proportion in the tumor. These stem cells are resistant to IFN, which could also explain the resistance to treatment [36]. Moreover, IFN-induced asthenia and daily subcutaneous administration may limit treatment observance.

Tyrosine-kinase inhibitors (TKIs) interact competitively with adenosine triphosphate to block phosphorylation of the intracellular tyrosine-kinase sites. The use of TKI is based on the overexpression of target proteins in tumors and their stroma. PDGFR is a receptor of the tyrosine-kinase family. After activation by its ligand (PDGF), it dimerizes and initiates a signaling cascade involving in particular the PI3K pathway, resulting in proliferation and cell differentiation. Vascular endothelial growth factor receptor (VEGFR), another tyrosine-kinase receptor, is a key pro-angiogenic factor. Type 1, 2, and 3 VEGFR are located on endothelial cells, and when activated, they cause the migration and proliferation of these cells. Proto-oncogene C-Kit (CD117), which belongs to the same family, links the stem cell growth factor and is a therapeutic target as well.

Several TKIs are available, and imatinib (Gleevec®) is the most commonly used in fibromatosis. It targets C-Kit, PDGF, and Bcr-Abl. Imatinib was not used in our case because it was reported as resistance phenomena [37]. Sorafenib (Nexavar®) is a multiple inhibitor of tyrosine kinase (C-Kit, PDGFR-β, VEGFR2-3). It can be administered orally. In a clinical study including 26 patients with fibromatosis (IAF and EAF, including six patients with a location in the trunk or chest), sorafenib was administered at a daily dose of 400 mg and showed benefits both clinically (6 months, improvement of symptoms) and radiologically (tumor stabilization or partial response) [38]. Based on these data, sorafenib treatment was pursued during 18 months. However, due to digestive and cutaneous adverse effects and tumor stagnation, sorafenib was replaced with sunitinib (Sutent®), another TKI (VEGFR, PDGFR, Kit, FLT3). A phase II clinical study has evaluated the effects of sunitinib in EAF and IAF. Of 19 patients, five had trunk fibromatosis: for an average treatment duration of 9.6 months, tumor progression was observed in one patient, stability in two patients, and partial response in one patient (one patient was not assessed) [39]. In our case, sunitinib was the only treatment that resulted in a significant partial response since a 50% and then 57% decrease in tumoral volume was observed since the introduction of this treatment, whereas the previous treatments failed to demonstrate tumor regression.

Our patient is now asking about a potential pregnancy. Breast fibromatosis affect young women, and pregnancy is a particularly problematic issue. A study of Fiore et al., including 92 pregnant patients suffering from fibromatosis, showed that in 48% of cases, the onset of fibromatosis was related to pregnancy: the diagnosis was made either during pregnancy or 6 months after childbirth. Otherwise, 52% of the patients already had a history of fibromatosis, which was either clinically evident during pregnancy or had appeared before but already treated. The risk of tumor progression is high during or after pregnancy, even for patients already treated [40]. Our patient is now aged 23. Sorafenib and sunitinib should not be used during pregnancy unless “absolutely necessary.” Animal studies have shown that they have teratogenic effects [41, 42]. To our knowledge, there are no reported cases of use of these substances in pregnant women. However, studies have shown an effect of imatinib on pregnancy: on 125 pregnancies occurred during treatment, 12 fetal malformations were observed, including three of a presumed specific pattern combining exomphalos to kidney and vertebral anomalies. Spontaneous miscarriage was observed in 18 women [43]. Other studies have shown meningocele [44], minor defects such as clinodactyly [45], and intrauterine growth restriction [46]. As our patient is now planning a pregnancy and the tumoral volume has reduced, we are currently considering stopping sunitinib and a resection of the tumor.

## Conclusions

As shown in our patient, medical treatments have heterogeneous efficacy. Targeted therapies may be a serious option to consider, especially when surgery is considered as high risk, thus leading to a less extensive surgery and a better functional and esthetic result. However, no pharmacological mechanism can unequivocally explain TKI’s efficacy. Besides medical treatments, other therapies are in development, such as cryotherapy. A trans-disciplinary approach is essential when dealing with desmoid-type mammary fibromatosis, joining targeted therapies to surgery/cryotherapy.

## Abbreviations

AF: Abdominal fibromatosis
CD: Cluster of differentiation
CK1γ: Casein Kinase 1 gamma
CNGOF: Collège National des Gynécologues Obstétriciens de France
COX: Cyclooxygenase
CTNNB1: Catenin Beta 1 gene
EAF: Extra-abdominal fibromatosis
ERs: Estrogen receptors
FLT3: Fms-like tyrosine kinase 3
GSK3: Glycogen synthase kinase 3
IFN: Interferon
IFNAR-1: Interferon-α/β receptor 1
JAK/STAT: Janus kinase/signal transducer and activator of transcription
LRP6: Low-density lipoprotein receptor-related protein 6
MRI: Magnetic resonance imaging
NSAIDs: Non-steroidal anti-inflammatory drugs
PDGFR: Platelet-derived growth factor (receptor)
PI3K: Phosphatidylinositol-4,5-bisphosphate 3-kinase
TKIs: Tyrosine-kinase inhibitors
VEGFR: Vascular endothelial growth factor (receptor)
WHO: World Health Organization
IAF: Intra-abdominal fibromatosis
